# A New Tool for *in vivo* Manipulation of Brain microRNA Levels: The Work of Smalheiser et al. (2014)

**DOI:** 10.3389/fpsyt.2015.00044

**Published:** 2015-04-20

**Authors:** Don A. Baldwin

**Affiliations:** ^1^Signal Biology, Inc., Philadelphia, PA, USA

**Keywords:** enoxacin, cortex, miRNA, rat learned helplessness, depression

Complex molecular networks often pose significant challenges for separating causes from effects, especially when using *in vivo* models to conduct experiments. Tackling such a challenge requires at least three (and usually more) critical tools: a means to reliably manipulate individual molecular factors, a measurable reporter with valid connections to the cell/tissue/organism behavior of interest, and an experimental system that allows repeated measurements over time. Smalheiser, Zhang, and Dwivedi describe a rat model that may provide these tools for studying molecular mechanisms of major depressive disorder (MDD).

Major depressive disorder encompasses a wide range of neurological symptoms, perhaps reflecting the wide variety of cellular and molecular alterations which have been reported in multiple brain regions (1). Smalheiser et al. have observed reduced microRNA expression levels in the prefrontal cortex of depressed suicide subjects (2, 3), but learned helplessness induced in a rat model of MDD was accompanied by muted responses in specific microRNAs compared to significantly reduced expression in rats that did not develop learned helplessness (4). As potent down-regulators of messenger RNA abundance and translation, microRNAs target a majority of genes in the human genome and thus represent a global, and potentially druggable (5, 6), regulatory mechanism capable of affecting most molecular networks. But do alterations in microRNA expression precede and potentially cause depression severe enough to result in suicide, comprise part of the response to some other triggering pathology, or represent both cause and effect depending on which microRNA is involved (7)?

Two factors that most influence microRNA expression levels are transcription regulation and RNA processing. microRNAs are short portions of sequence cleaved from longer precursor transcripts. Stabilization of the Dicer complex, the enzyme responsible for transcript cleavage to produce mature microRNAs, will result in higher levels of microRNA as long as unprocessed precursor transcripts are available. This effect can be achieved by supplying enoxacin, a fluoroquinolone that binds to a member of the Dicer complex and increases overall microRNA levels in cultured cells (8). Fluoroquinolones are an interesting family of small molecules that exhibit a number of bioactive properties; enoxacin not only interacts with TAR RNA-binding protein 2 (TARBP2) to promote Dicer activity but also alters V-ATPase binding to actin (9), JNK signaling (10), and cytochrome P450 activities (11–13), as well as inhibiting prokaryotic DNA gyrases (14).

Smalheiser et al. hypothesized that boosting microRNA abundances, or at least preventing their reduction, may disrupt the onset of learned helplessness in the rat MDD model, but it was not previously known whether enoxacin would be available to or active in the brain. Their current work demonstrates that 1 week of enoxacin exposure indeed does raise the levels of four neuronal reporter microRNAs in rat frontal cortex compared to unexposed controls. In parallel experiments, inescapable shock induced learned helplessness in 6 of 10 untreated rats, but only of 5 of 34 rats pretreated with enoxacin developed learned helplessness. These findings establish an experimental system that can now be used for a variety of follow-up studies using the three key tools – enoxacin to manipulate brain microRNA, learned helplessness as the reporter measurement, and permutations of timing and dosage to investigate time courses of causes and effects. A potentially useful addition would be assays of blood microRNA as a surrogate for brain expression levels, allowing repeated testing of animals without sacrificing to collect brain tissue; circulating microRNAs are becoming a well-established class of biomarkers for several pathologies including traumatic brain injury (15). Important topics to be addressed include the efficacy of enoxacin treatment after the onset of learned helplessness, and brain RNA and protein profiling to catalog the affected microRNAs, genes, and anatomical regions. As with any model system several caveats must be considered, such as whether the anti-depressive effect is due to enoxacin interaction with some other protein rather than stabilization of Dicer, and how well this rat model represents human MDD at brain biochemical up through behavioral levels.

As a bacterial DNA gyrase inhibitor, enoxacin has been successfully used worldwide as a second-generation quinolone antibiotic with low occurrence of adverse effects, although a perception of higher toxicity compared to related compounds has limited its use in the United States. Perhaps fortelling the cortical microRNA result, enoxacin has been associated with central nervous system effects including insomnia, photosensitivity, and convulsions (when combined with NSAIDs) (16, 17). Interest in enoxacin has been renewed by cancer microRNA profiling, which revealed wide-scale reductions in expression, including the loss of many tumor-suppressor microRNAs. Barring somatic mutation of TARBP2 that would prevent its binding, enoxacin can alleviate suppressed microRNA production and restore molecular controls that normally prevent tumorigenesis or send neoplastic cells into cell cycle arrest or apoptosis (6, 8, 18, 19). Reduced microRNA production is associated with amyotrophic lateral sclerosis and other neurodegeneration (7, 20–24), and enoxacin is therefore under consideration as a treatment for motor neuron diseases (25). Another potential therapeutic application (likely not involving microRNA) is inhibition of osteoclast development and activity, which could prevent damaging bone resorption in periodontal disease and around orthopedic implants that lose stability with wear (9, 10, 26, 27). Would MDD patients, who may need a much longer course of enoxacin exposure than is typical for antibiotic indications, experience relief of depression and protection against cancer at the expense of excessive bone deposition and an altered microbiome? At a minimum, enoxacin provides a well-tolerated tool for *in vivo* investigation of how microRNA processing affects brain phenotypes (28), and a starting point for identifying specific therapeutic targets that could be treated with synthetic microRNAs or their antisense inhibitors (29–31).

## Conflict of Interest Statement

The author declares that the research was conducted in the absence of any commercial or financial relationships that could be construed as a potential conflict of interest.
